# Coexistence of a nonresistance-conferring IncI1 plasmid favors persistence of the *bla*_CTX-M_-bearing IncFII plasmid in *Escherichia coli*

**DOI:** 10.1128/spectrum.04240-23

**Published:** 2024-04-30

**Authors:** Kun He, Jiayu Lin, Yulei Liang, Junling Cui, Qiuru Chen, Yanbin Dong, Xiaoyuan Ma, Dandan He, Li Yuan

**Affiliations:** 1 College of Veterinary Medicine, Henan Agricultural University, Zhengzhou, China; 2 International Joint Research Center of National Animal Immunology, College of Veterinary Medicine, Henan Agricultural University, Zhengzhou, China; 3 Key Laboratory of Quality and Safety Control of Poultry Products (Zhengzhou), Ministry of Agriculture and Rural Affairs, Zhengzhou, China; 4 Zhengzhou Key Laboratory of Research and Evaluation of Traditional Chinese Veterinary Medicine, Zhengzhou, China; Texas A&M University, College Station, Texas, USA

**Keywords:** coinhabitant plasmids, *bla*_CTX-M_-IncFII, nonresistance-IncI1, fitness advantage, plasmid persistence

## Abstract

The interaction between coexisting plasmids can affect plasmid-carried resistance gene persistence and spread. However, whether the persistence of the *bla*_CTX-M_ gene in clinical Enterobacteriaceae is related to the interaction of coresident nonresistance-conferring plasmids has not been reported. This study was initiated to elucidate how a nonresistance-conferring IncI1 plasmid affected the *bla*_CTX-M_-bearing IncFII plasmid colocated on the same cell. Herein, we constructed three isogenic derivatives of *E. coli* C600, designated as C600_FII_, C600_I1_, and C600_FII+I1_, which harbored the *bla*_CTX-M_-IncFII plasmid and/or the nonresistance-IncI1 one. We discovered that strain C600_FII+I1_ conferred higher fitness advantages than strain C600_FII_; also, the stability of the *bla*_CTX-M_-IncFII plasmid was noticeably improved in an antibiotic-free environment when it coexisted with the IncI1 plasmid. To further explore why the IncI1 plasmid enhanced the persistence of the *bla*_CTX-M_-IncFII plasmid, we assessed the *bla*_CTX-M_-IncFII plasmid's copy numbers, conjugation frequencies, and *rep* gene expressions in strains C600_FII_ and C600_FII+I1_. The results demonstrated that the *rep* expressions of the *bla*_CTX-M_-IncFII plasmid in strain C600_FII+I1_ was greatly decreased, along with the plasmid’s copy numbers and mating efficiencies, compared to those in strain C600_FII_. Moreover, further study revealed that the intracellular ATP levels of strain C600_FII+I1_ were far lower than those of strain C600_FII_. Our findings confirmed that coexistence of the nonresistance-IncI1 plasmid can keep the *bla*_CTX-M_-IncFII plasmid more stable by increasing the fitness advantages of the host bacteria, which will pose a threat to preventing the long-term presence of the plasmid-carried *bla*_CTX-M_ gene in clinical Enterobacteriaceae.

**IMPORTANCE:**

So far, plasmid-carried *bla*_CTX-M_ is still the most common extended-spectrum beta-lactamase (ESBL) genotype in clinical settings worldwide. Except for the widespread use of third-generation cephalosporins, the interaction between coexisting plasmids can also affect the long-term stable existence of the *bla*_CTX-M_ gene; however, the study on that is still sparse. In the present study, we assess the interaction of coinhabitant plasmids *bla*_CTX-M_-IncFII and nonresistance-IncI1. Our results confirmed that the increased fitness advantages of strain C600_FII+I1_ were attributable to the cohabitant nonresistance-IncI1 plasmid, which largely reduced the intracellular ATP levels of host bacteria, thus decreasing the rep gene expression of the *bla*_CTX-M_-IncFII plasmid, its copy numbers, and mating efficiencies, while the higher fitness advantages of strain C600_FII+I1_ enhanced the persistence of the *bla*_CTX-M_-IncFII plasmid. The results indicate that the nonresistance-IncI1 plasmid contributes to the long-term existence of the *bla*_CTX-M_-IncFII plasmid, implying a potentially new strategy for controlling the spread of resistance plasmids in clinical settings by targeting nonresistance plasmids.

## INTRODUCTION

The production of extended-spectrum beta-lactamases (ESBLs) is the main factor leading to the resistance of Gram-negative bacteria to β-lactam antibiotics (1). Until now, *bla*_CTX-M_ has emerged as the most prevalent ESBL genotype in clinical Enterobacteriaceae worldwide (2). Conjugative plasmids, specifically IncFII-type plasmids, have a crucial role in the development and horizontal dissemination of *bla*_CTX-M_ (3). IncFII plasmids are widely distributed in Enterobacteriaceae, such as in *E. coli*, (4) and they help the host bacteria adapt to various environments, conditions, and pressures (5, 6). Although resistance plasmids confer potential benefits to the host, they also impose burdens (fitness costs), manifesting as reduced growth rates and competitiveness of strains carrying plasmids under conditions where plasmid-encoded resistance genes are not selected (7), while the increased cost of plasmid adaptation and the potential loss of plasmids during bacterial cell division severely reduce plasmid persistence (7). In addition, plasmid–host genome interactions also affect the evolution of plasmid persistence (8).

Recent studies suggested that a sufficiently high transfer rate could appropriately compensate for the adaptive cost and plasmid loss (9, 10). Therefore, suppressing conjugation for disrupting the persistence of resistance plasmids is an attractive and effective strategy. Many factors, such as bacterial genetic backgrounds, plasmid copy numbers, and interactions with other resistance plasmids, could affect the conjugation of antibiotic resistance plasmids (11, 12). For example, Haudiquet *et al*. reported that conjugation rates depend on the capsule serotype of both donor and recipient strains. (13). Meanwhile, increasing evidence suggests that the interactions of multiple resistance plasmids could change the persistence of plasmid-harbored resistance genes (14, 15). Gama *et al*. found that interactions between plasmids could determine the mode of antibiotic resistance dissemination in bacterial populations (16, 17), while Dionisio and his team demonstrated that the transfer rate of plasmids was affected by the presence of different plasmids in recipient cells (18). However, it has not been reported whether the persistence of the *bla*_CTX-M_ gene in clinical *E. coli* is related to the interaction of coinhabitant nonresistance plasmids.

In the survey of antimicrobial-resistant bacterial isolates in 2021, *E. coli* LWY24 was reported from feces of healthy chicken in Henan Province, China (19). The isolate LWY24 harbored three conjugative plasmids, pLWY24J-3 (*bla*_CTX-M-55_-bearing IncFII-type, MN702385), pLWY24J*mcr-1* (*mcr-1*-carrying IncI2-type, MN689940), and pLWY24J-4 (nonresistance-conferring IncI1-type), which could transfer horizontally through plasmid conjugation independently, in pairs or three together. In 2022, we investigated the interaction between the *bla*_CTX-M_-bearing IncFII plasmid and the *mcr-1*-carrying IncI2 plasmid coexisting in the same strain (20). The findings confirmed that coresident IncFII-type and IncI2-type plasmids in *E. coli* were stably persistent, conferred more fitness advantages, and were easier to transfer and cotransfer. In addition, it was discovered that the mating efficiency of the *bla*_CTX-M_-IncFII plasmid decreased by 3.467 × 10^4^ fold when coexisting with the nonresistance-IncI1 plasmid. However, the correlation between the two plasmids remains unclear. This study was initiated to explore the underlying mechanism that the nonresistance-conferring IncI1 plasmid affected the *bla*_CTX-M_-bearing IncFII plasmid colocated on the same bacterial host.

## RESULTS AND DISCUSSION

### The plasmids had no effect on bacterial growth

**Fig 1:**
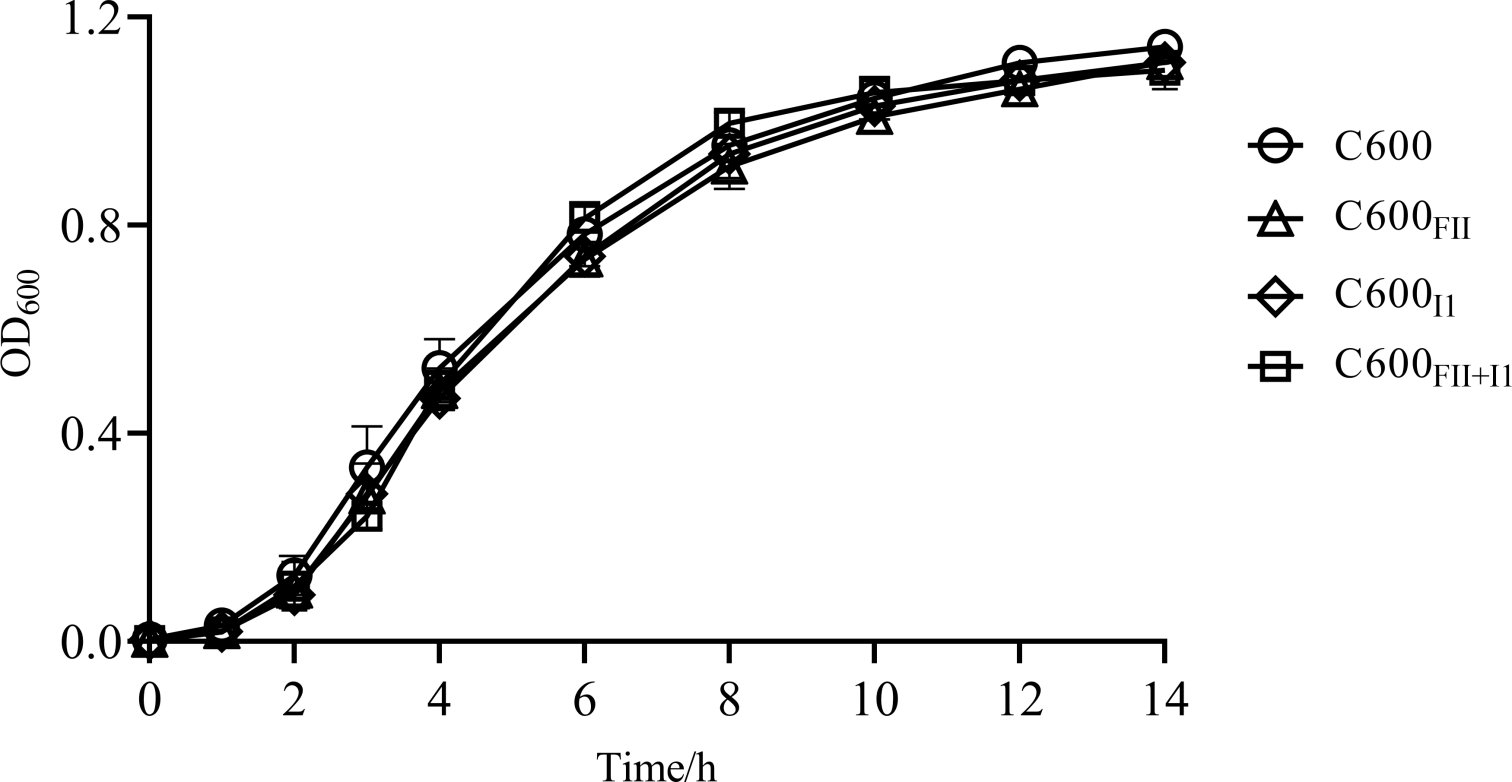
Growth curves of *E. coli* C600 and its isogenic derivatives, C600_FII_, C600_I1_, and C600_FII+I1_. The strains C600_FII_, C600_I1_, and C600_FII+I1_ were isogenic derivatives of *E. coli* C600, which harbored the plasmids *bla*_CTX-M_-IncFII and/or nonresistance-IncI1. Curves indicated the mean of results from three independent experiments, and error bars denoted standard deviations for each timepoint.

To explore the effects of plasmids FII and I1 on bacterial growth, we plotted the growth curves of strains *E. coli* C600, C600_FII_, C600_I1_, and C600_FII+I1_ based on the optical density at 600 nm (OD_600_) values after 14-h assessment (Fig. 1). The results showed that there were no significant differences in growth among bacteria, indicating that the plasmids FII and/or I1 did not impair the growth of the bacterial host.

### Plasmid I1 improved the stability of the coexisting plasmid FII

**Fig 2:**
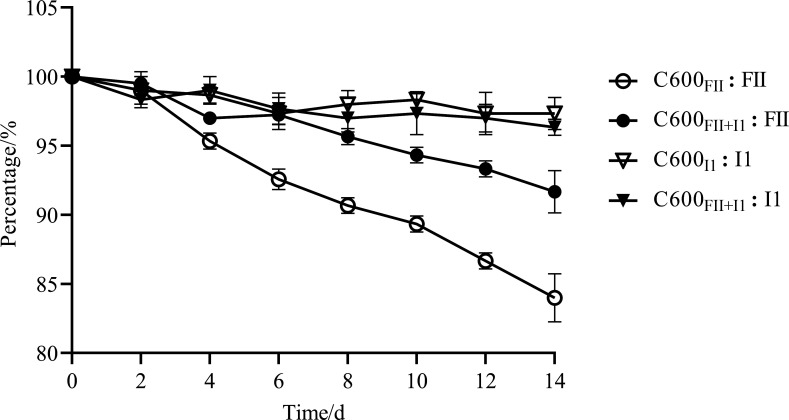
The stability of plasmids IncFII and IncI1 in strains C600_FII_, C600_I1_, and C600_FII+I1_. The strains C600_FII_, C600_I1_, and C600_FII+I1_ were isogenic derivatives of *E. coli* C600, which harbored the plasmids *bla*_CTX-M_-IncFII and/or nonresistance-IncI1. Data shown are the means of results from three independent assays, and error bars represented the standard deviation of the mean (*n* = 3).

When multiple plasmids are present in the same bacterium, interactions between them can affect their transmission and persistence (15, 21). In order to plot the stability–time curve of the acquired plasmids, we propagated the isogenic strains, C600_FII_, C600_I1,_ and C600_FII+I1_ culture, in antibiotic-free medium for 2 weeks (Fig. 2). The results showed that the single plasmid FII was moderately stable, remaining at around 84% on day 14, which coincided with our previous findings (20). However, when plasmids FII and I1 cohabited in the recipient cell, the stability of plasmid FII was obviously improved, with a total loss of about 8.0% (*P* = 0.0047) at the end, implying that plasmid I1 could improve the persistence of the coexisting plasmid FII.

### Coharboring plasmids FII and I1 endow cells with fitness advantages

**Fig 3:**
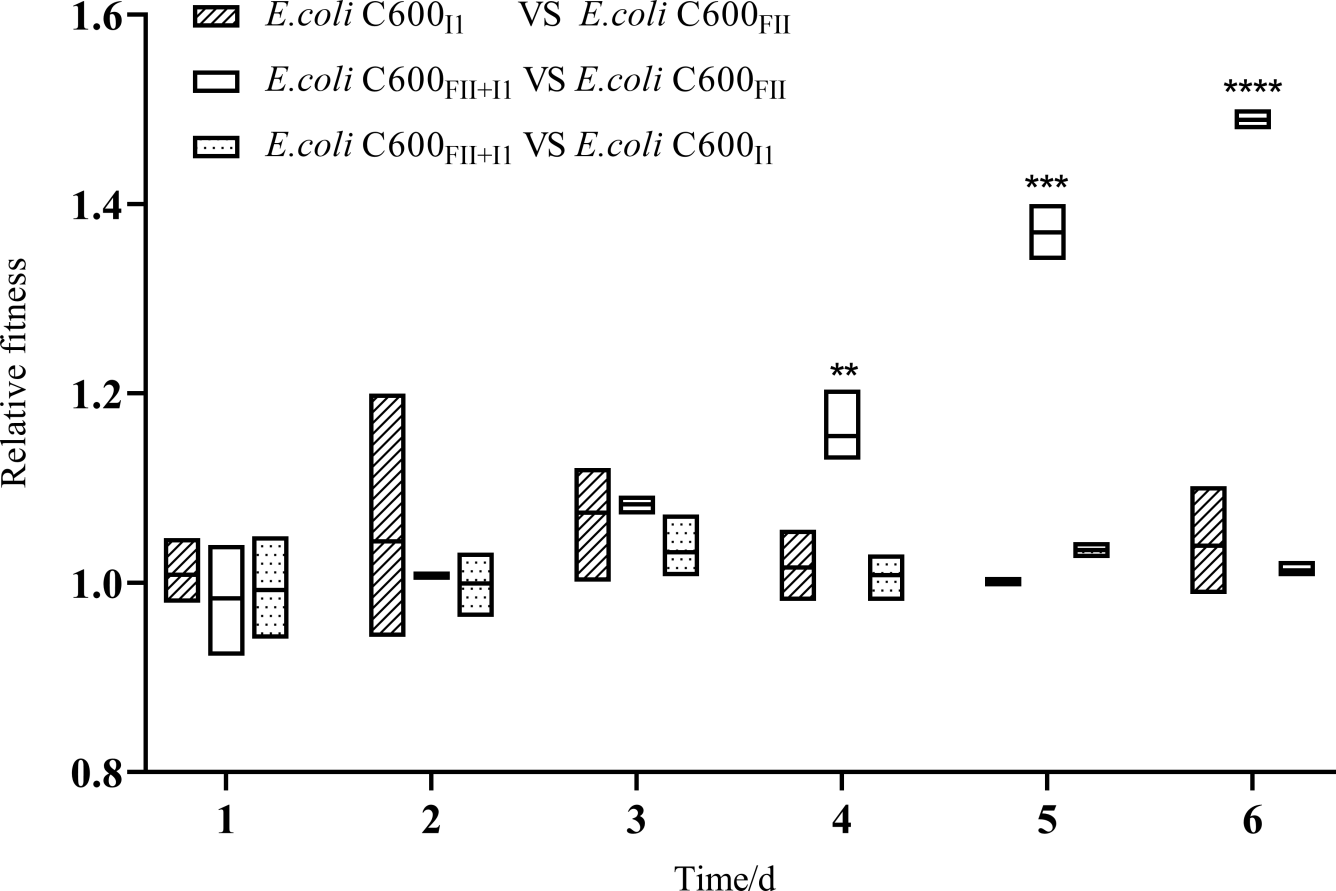
Pairwise competition assays between strains C600_FII_, C600_I1_, and C600_FII+I1_. The strains C600_FII_, C600_I1_, and C600_FII+I1_ were isogenic derivatives of *E. coli* C600, which harbored the plasmids *bla*_CTX-M_-IncFII and/or nonresistance-IncI1. Each boxplot represents the distribution of relative fitness values for each timepoint: the horizontal line in the boxplot is the median, and the bottom and top of the boxplot show the lowest and the highest values, respectively. Asterisks denote significant differences using unpaired *t* test (*P < 0.05; **P < 0.01; ***P < 0.005).

To determine the relative carriage costs of plasmids FII and I1, competition assays *in vitro* were carried out among strains C600_FII_, C600_FII+I1,_ and C600_I1_ (Fig. 3). The outcome revealed that there was no significant difference in the fitness costs between strains C600_FII_ and C600_I1_ as well as between strains C600_FII+I1_ and C600_I1_, indicating that plasmid FII would not endow any additional fitness costs with its host cell when transferring to strain C600_I1_. On the contrary, the isogenic strain C600_FII+I1_ significantly outcompeted C600_FII_ from day 4 (RF = 1.155 ± .035, *P* = 0.0148) to day 6 (RF = 1.489 ± .011, *P* = 0.0001), indicating that plasmid I1 could increase the fitness advantages of host bacteria when entering strain C600_FII_. Thus, *E. coli* co-carrying plasmids FII and I1 present higher fitness advantages than bacteria carrying single plasmid FII, which helps the *bla*_CTX-M_-IncFII plasmid maintain long-term persistence.

### The reduced copies of plasmid FII were due to the coexistence of plasmid I1

To verify whether copy numbers of plasmid FII in strain C600_FII+I1_ decreased, we conducted qPCR according to our previous research (20). The findings exhibited that the copy numbers of plasmid FII in strain C600_FII+I1_ were 0.467 ± 0.09 per cell (Table 1), which were extremely reduced compared to those in strain C600_FII_ (1.773 ± 0.16 per cell) (*P* < 0.001), implying that plasmid I1 inhibited coexisting plasmid FII copies. Generally, the entry of plasmids into new bacterial hosts via conjugative transmission can increase fitness costs (22). Therefore, for long-term survival in the environment, plasmids have evolved corresponding adaptive compensation mechanisms to compensate for the increased costs caused by plasmid entry and expression (7). Previous reports have proven that reducing plasmid copies is the best way to directly reduce the costs of survival (9). In the present study, the copy numbers of plasmid FII were dramatically reduced under the action of the coexisting plasmid I1, thereby increasing the fitness advantages of bacterial hosts and enhancing the persistence of the *bla*_CTX-M_-IncFII plasmid.

**TABLE 1:** The plasmid copy numbers in strains C600_FII_, C600_I1,_ and C600_FII+I1_^*a*^

Strains	Plasmids	Plasmid copy numbers (mean ± standard deviation)
C600_FII_	IncFII	1.773 ± 0.16^a^
C600_I1_	IncI1	1.157 ± 0.12
C600_FII+I1_	IncFII	0.467 ± 0.09^b^
	IncI1	1.039 ± 0.05

^*a*^ All data were obtained from at least three biological replicates and presented as mean ± SD. Different letters marked with the shoulder mark in the same column indicate significant differences (a vs b, *P* < 0.001). The strains C600_FII_, C600_I1_, and C600_FII+I1_ were isogenic derivatives of *E. coli* C600, which harbored the plasmids *bla*_CTX-M_-IncFII and/or nonresistance-IncI1.

### Plasmid I1 depressed coresident plasmid FII transfer

The conjugation rates of plasmid FII were measured using C600_FII_ and C600_FII+I1_ as a donor and *E. coli* J53 as a recipient (Table 2). The mating rate of plasmid FII in the donor C600_FII_ was (9.650 ± 3.437) ×10^−2^, which was consistent with the findings in our previous study (20). Moreover, in the donor C600_FII+I1_, two types of transconjugants can be isolated on MacConkey agar containing cefotaxime, one harboring only a single plasmid FII at (1.869 ± 0.294)×10^−6^ per donor cell and the other co-carrying plasmids FII and I1 with frequencies of (9.195 ± 4.065)×10^−7^. In total, the conjugation rates of plasmid FII in the donor C600_FII+I1_, including independent transfer and co-transfer with plasmid I1, were (2.783 ± 0.288) ×10^−6^ per donor cell, which were approximately 3.467 × 10^4^-fold lower than those in the donor C600_FII_. Thus, the horizontal transfer of plasmid FII can be severely inhibited by plasmid I1 coexisting in the same bacterial host.

**TABLE 2:** The transfer rates of the IncFII-type plasmid in strains C600_FII_ and C600_FII+I1_^*a*^

Donor	Recipient	Transconjugant	Transfer rates (mean ± standard deviation)
C600_FII_	*E. coli* J53	J53_FII_	(9.650 ± 3.437) ×10^-2 a^
C600_FII+I1_		J53_FII_ & J53_FII+I1_	(2.783 ± 0.288) ×10^-6 b^
		J53_FII_	(1.869 ± 0.294) ×10^−6^
		J53_FII+I1_	(9.195 ± 4.065) ×10^−7^

^*a*^ Experiments were performed three times, and results are shown as mean  ±  SD. Different letters marked with the shoulder mark in the same column indicate significant differences (a vs b, *P* < 0.001). The strains C600_FII_ and C600_FII+I1_ were isogenic derivatives of *E. coli* C600, which harbored the single *bla*_CTX-M_-IncFII plasmid or coinhabitant plasmids *bla*_CTX-M_-IncFII and nonresistance-IncI1.

Usually, high mating efficiencies contribute to plasmid persistence, but increase plasmid fitness costs (22). However, in our study, the mating efficiency of plasmid FII in strain C600_FII+I1_ sharply decreased, compared to that of strain C600_FII_, although it presented more stable. Gama *et al.* reported that those affecting fitness favor plasmid persistence more than those affecting conjugative transfer (14). Stevenson *et al*. also proved that in environments that experience occasional pulses of positive selection, even low rates of conjugation can enhance plasmid survival by enabling low-level persistence during the periods between pulses (23). Thus, we speculate that the lower plasmid conjugation rates of strain C600_FII+I1_ contribute to the fitness advantages of bacterial hosts, thereby improving the persistence of plasmid FII.

### The invasion of plasmid I1 enhances a long-time persistence of strain C600_FII+I1_

**Fig 4:**
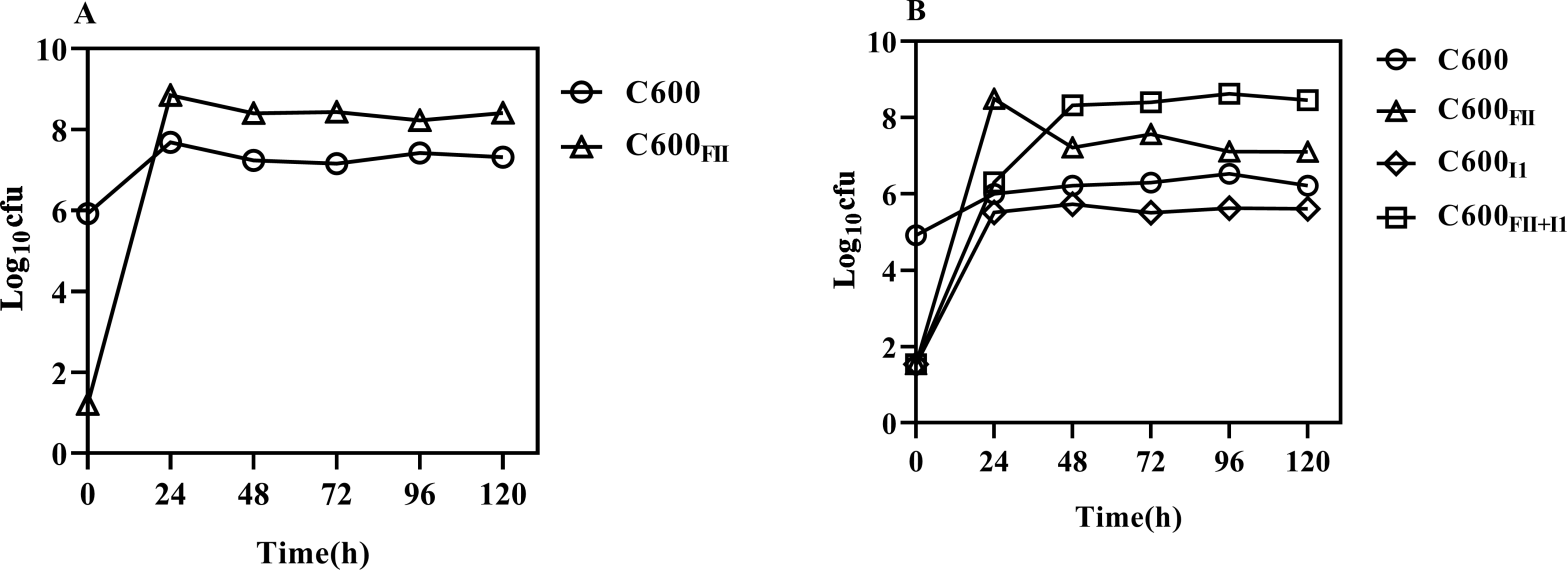
Plasmid invasion assays of plasmids *bla*_CTX-M_-IncFII and IncI1. (**A**) Cocultures with *E. coli* C600 and C600_FII_. (**B**) Cocultures with *E. coli* C600 and C600_FII+I1_. Calculate viable bacteria of co-cultures with plasmid-free and plasmid-containing *E. coli* C600. Plasmid-free *E. coli* were mixed with a 10^−3^-fold excess of plasmids containing single plasmid *bla*_CTX-M_-IncFII and colocated plasmids *bla*_CTX-M_-IncFII and IncI1 at the beginning of the invasion assay. Every dot indicated the average value of three biological replicates. *E. coli* C600 is represented by hollow circles, C600_FII_ is represented by hollow triangles, C600_I1_ is represented by hollow diamonds, and C600_FII+I1_ is represented by hollow squares.

Plasmid persistence could comprehensively be evaluated by comparing the abilities of single-plasmid and co-located plasmids in invading plasmid-free populations (24). As shown in Fig. 4A, plasmid FII could invade rapidly and appeared in most cells after 24 h, and its numbers not only remained unchanged over time but were significantly higher than those of plasmid-free bacteria. Considering that the individual plasmid FII was slightly unstable, we speculated that the higher mating efficiency of plasmid FII could help offset the loss of plasmid FII. Interestingly, when *E. coli* C600 and C600_FII+I1_ were coincubated, plasmids FII and I1 could successfully invade bacteria either independently or together, thereby obtaining three types of plasmid-harbored bacteria, C600_FII_, C600_I1,_ and C600_FII+I1_ (Fig. 4B). Specifically, when cultured for 24 hours, strain C600_FII_ was 2.91 × 10^8^ CFU/mL, C600_FII+I1_ was 2.05 × 10^6^ CFU/mL, while strain C600_I1_ was the least at that time, only 3.34 × 10^5^ CFU/mL, indicating the lowest invasion rates of plasmid I1. It was worth noting that strain C600_FII_ decreased extremely during the incubation period of 24 h to 48 h; on the contrary, strain C600_FII+I1_ was found to apparently increase, implying that plasmid I1 could continue to invade strain C600_FII_. Further study showed that C600_FII+I1_ exhibited stability in the mixed populations after 48 hours of culture, which was consistent with the aforementioned results of plasmid stability assays. Given its lower plasmid conjugation rates, we speculated that the improved persistence of strain C600_FII+I1_ was mainly attributable to the invasion of plasmid I1, which enhanced its fitness advantages.

### Coexistence of plasmids FII and I1 did not change the swimming motility of the bacterial host

**Fig 5:**
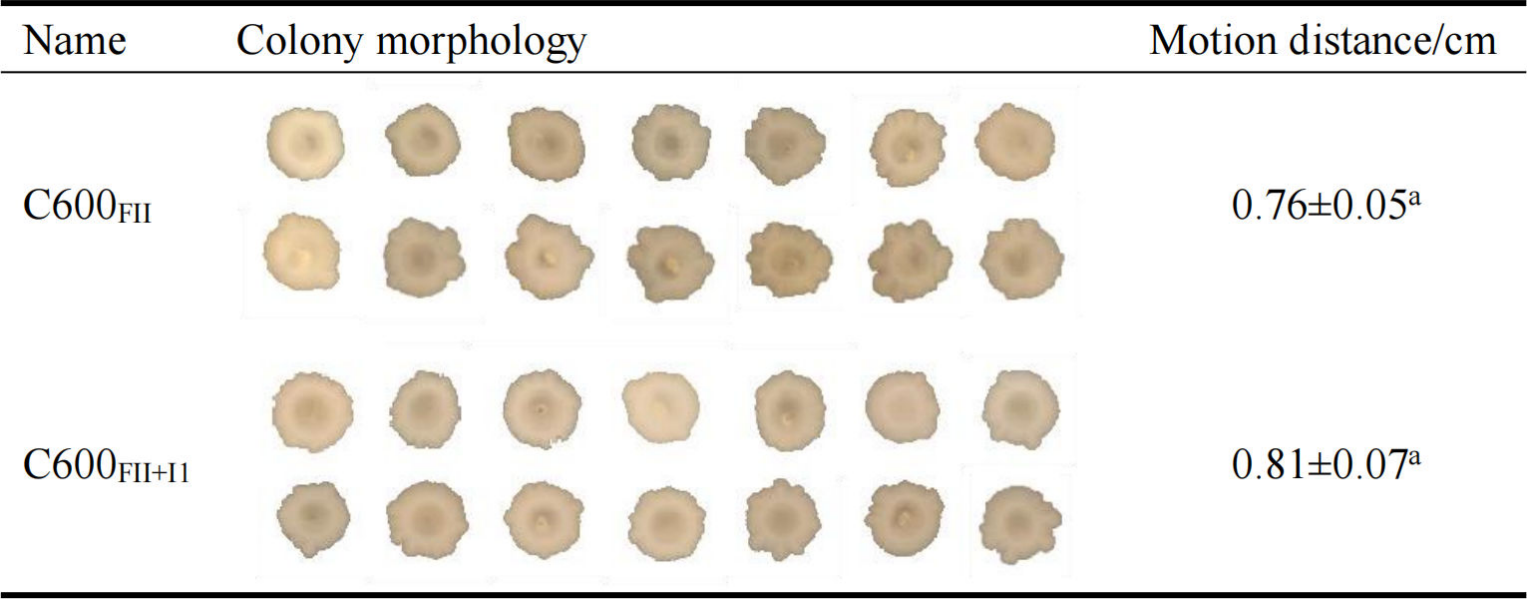
The swimming motility of strains C600_FII_ and C600_FII+I1_. Experiments were performed three times, and the results are shown as mean  ±  SD. “a” indicates that there is no significant difference in swimming mobility between strains C600_FII+I1_ and C600_FII_. The strains C600_FII_ and C600_FII+I1_ were isogenic derivatives of *E. coli* C600, which harbored the single *bla*_CTX-M_-IncFII plasmid or coinhabitant plasmids *bla*_CTX-M_-IncFII and nonresistance-IncI1.

It is generally believed that close contact is required between the donor and recipient before plasmid conjugative transmission; therefore, changing the bacterial motility characteristics can affect the transfer frequency of plasmids (25). The swimming motility assay was fulfilled in the isogenic strains C600_FII_ and C600_FII+I1_ (Fig. 5). Obviously, both strains C600_FII_ and C600_FII+I1_ could form an initial-inoculation-point-centered approximate circle on the swimming plates, and there was no available difference in their swimming motility. From this, we speculated that the inhibition of coexisting plasmid I1 on plasmid FII transfer was not achieved by affecting the motility of the bacterial host.

### Plasmid I1 decreased the *rep* gene expressions of co-resident plasmid FII

**Fig 6:**
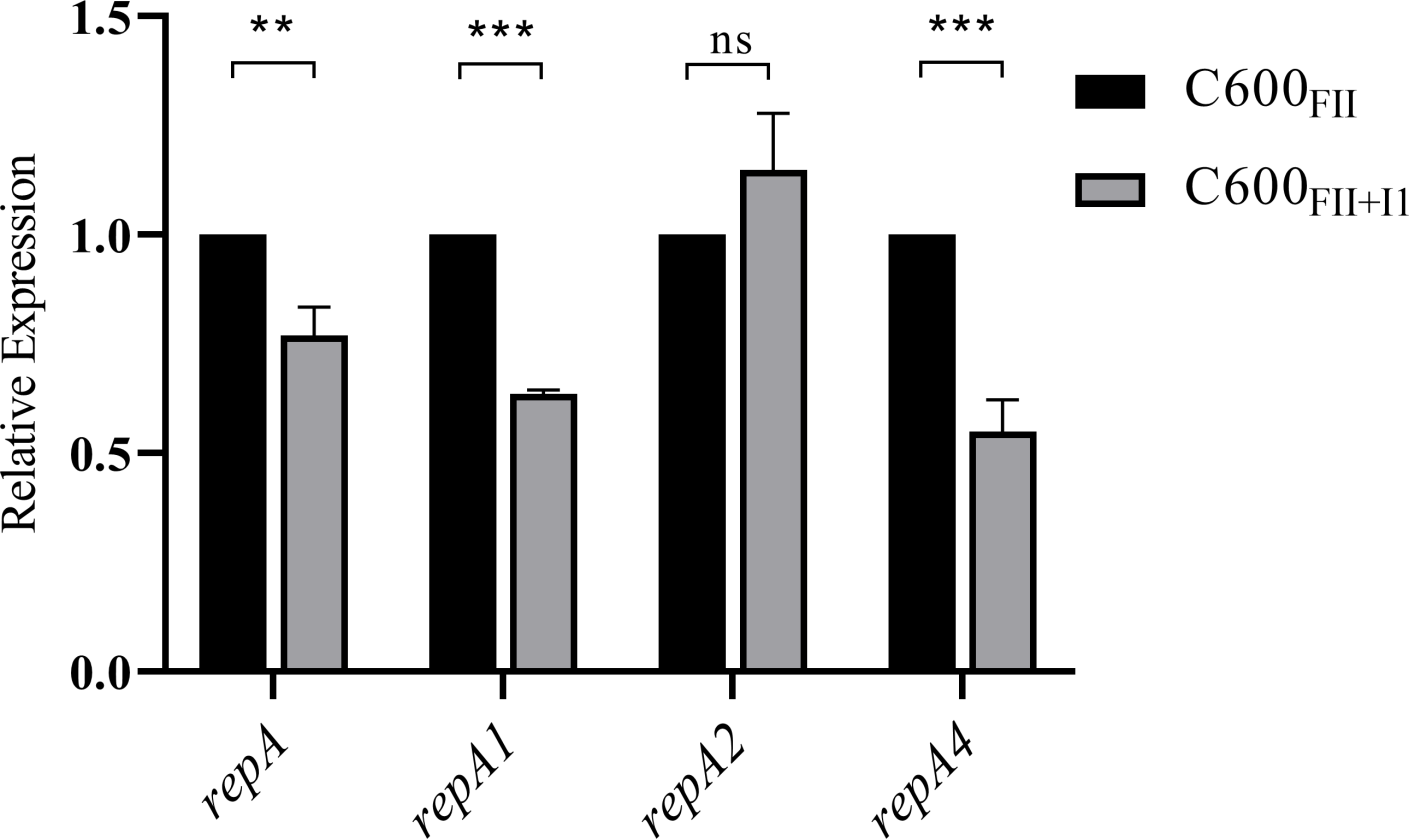
Expression levels of *rep* genes of the *bla*_CTX-M_-IncFII plasmid in strains C600_FII_ and C600_FII+I1_ by real-time relative quantitative PCR. The strains C600_FII_ and C600_FII+I1_ were isogenic derivatives of *E. coli* C600, which harbored the single *bla*_CTX-M_-IncFII plasmid or coinhabitant plasmids *bla*_CTX-M_-IncFII and nonresistance-IncI1. All data are presented as mean  ±  SD and analyzed by unpaired *T* test (**P < 0.01; ***P <  0.005; ****P < 0.001).

To determine whether the reduced conjugation rate of plasmid FII in strain C600_FII+I1_ was mediated by changes in *rep* gene expression levels, we examined the mRNA expression levels of *repA*, *repA1*, *repA2,* and *repA4* encoded by the plasmid FII (MN702385) (19). Except for *repA2*, the levels of *repA*, *repA1*, and *repA4* in strain C600_FII+I1_ were remarkably decreased (*P* ≤ 0.0036), compared to those of strain C600_FII_ (Fig. 6). Rep protein is a plasmid-encoded replication initiator, which is essential and rate-limiting for plasmid replication initiation and conjugative mobilization (26). Therefore, the inhibition of coexisting plasmid I1 on plasmid FII transfer is closely related to its repression of *rep* gene expressions in plasmid FII.

### The intracellular ATP levels decreased in strain C600_FII+I1_

**Fig 7:**
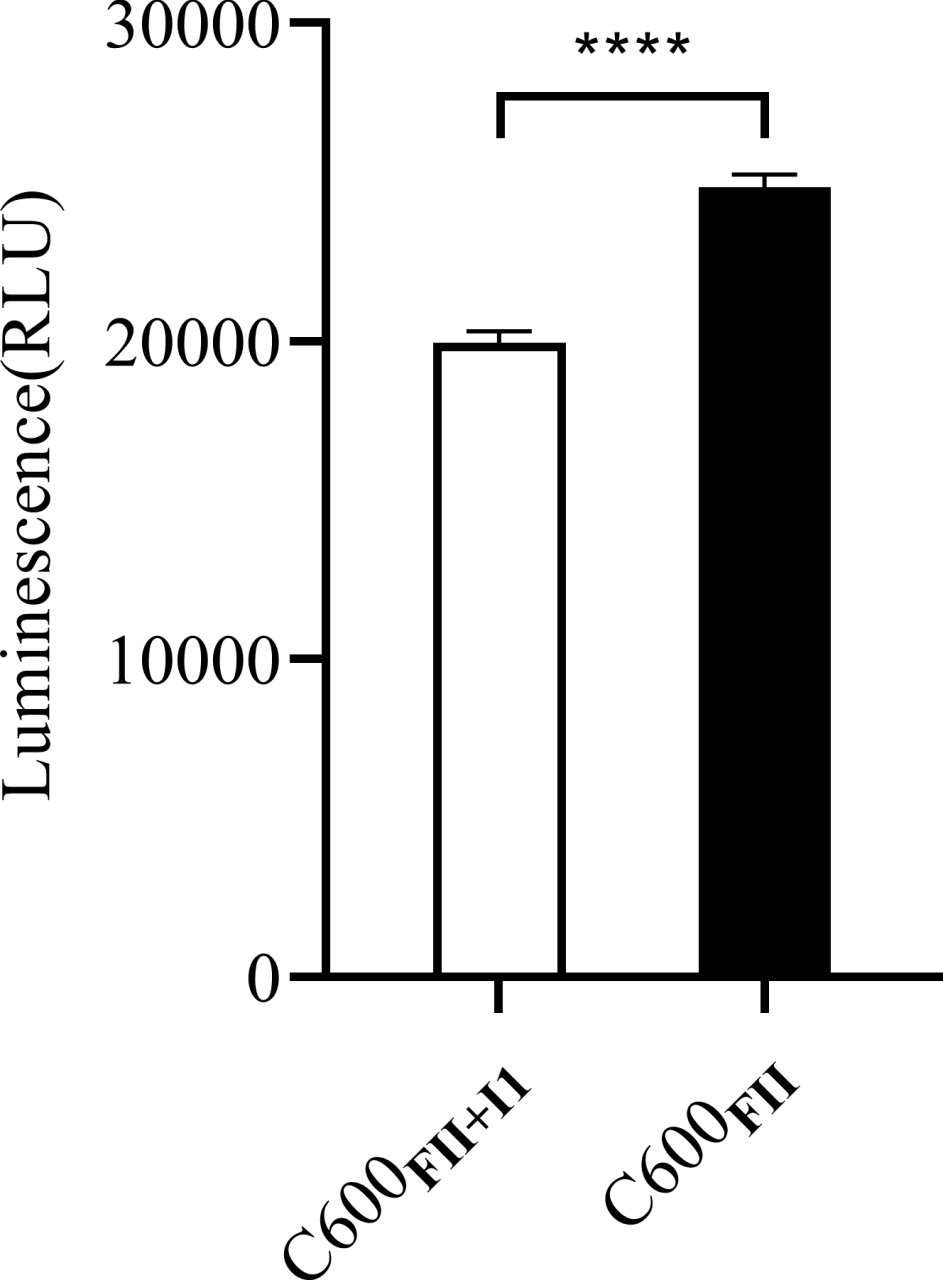
The intracellular ATP levels of the isogenic derivatives C600_FII_ and C600_FII+I1_. The strains C600_FII_ and C600_FII+I1_ were isogenic derivatives of *E. coli* C600, which harbored the single *bla*_CTX-M_-IncFII plasmid or coinhabitant plasmids *bla*_CTX-M_-IncFII and nonresistance-IncI1. Compared to C600_FII_, strain C600_FII+I1_ had significantly lower ATP levels (*P* < 0.001). All data were obtained from at least three biological replicates and presented as mean ± SD. Significance levels are indicated by the numbers of asterisks (****P < 0.001).

Conjugative plasmids in *E. coli* are transferred from one bacterium to another through a membrane-associated macromolecular channel called the type IV secretion system (T4SS) (21). ATP plays an important role in conjugation by providing energy for T4SS assembly and substrate transport, plasmid replication, and transport (27, 28). So we further measured the intracellular ATP levels in strains C600_FII+I1_ and C600_FII_. As expected, the fluorescence intensities in strain C600_FII+I1_ were markedly reduced, compared to strain C600_FII_ (*P* < 0.0001) (Fig. 7), indicating that the intracellular ATP levels of strain C600_FII+I1_ sharply decreased. Thus, we hypothesized that the decrease of intracellular ATP levels may be one of the reasons for the decrease of *rep* gene expression and mating efficiency of plasmid FII in strain C600_FII+I1_.

### Conclusion

Taken together, the higher fitness advantages of strain C600_FII+I1_ enhance its harboring *bla*_CTX-M_-IncFII plasmid persistence. While more fitness advantages of strain C600_FII+I1_ are attributable to the cohabitant nonresistance-conferring IncI1 plasmid that largely reduces the copies and mating efficiencies of the *bla*_CTX-M_-IncFII plasmid by inhibiting its *rep* gene expression, which was repressed by decreasing the intracellular ATP levels of host bacteria. Our findings demonstrate that the advantages are evident in the coexistence of plasmids *bla*_CTX-M_-IncFII and nonresistance-conferring IncI1 in *E. coli*, which can advance the long-term clinical presence of the *bla*_CTX-M_ resistance gene and pose a threat to controlling its spread.

In the past, the interaction between plasmids mainly focused on the coexistence of resistant plasmids (16, 18) and almost ignored whether nonresistance plasmids would have an effect on the co-inhabitant resistance one. In the present study, we found that the nonresistance IncI1 plasmid could improve the cohabitant *bla*_CTX-M_-IncFII plasmid fitness advantages and persistence by reducing its copies and mating efficiencies. As a result, it may be a potential new strategy that influencing resistance plasmid persistence in clinical settings by targeting certain nonresistance plasmids in the future.

## MATERIALS AND METHODS

### Plasmids, bacterial strains, and primers

*E. coli* C600_FII_, C600_I1,_ and C600_FII+I1_ harboring pLWY24J-3 (*bla*_CTX-M-55_-bearing, 68.72 kb, IncFII, F33:A-:B-, abbreviation for FII), pLWY24J-4 (nonresistance-conferring, 89.07 kb, IncI1, abbreviation for I1) or all of them, were obtained by conjugation from *E. coli* LWY24 O3:H25, ST93 isolated from a multidrug-resistant chicken in China (13). The primers used in this study are listed in Table 3, which were synthesized by Beijing Qingke Biotechnology Co., Ltd.

**TABLE 3:** The primers used in the study

Target gene	Primer sequences (5’ to 3’)	Length/bp	Reference/ GeneBank
PCR primers for verifying and cloning *bla*_CTX-M_, *nusG* and *dxs* genes			
*bla*_CTX-M_	F: GCTCTAGATTACAAACCGTCGGTGAC	876	This study
	R: CGGAATTCATGGTTAAAAAATCACTG		
*nusG*	F: GCTCTAGACTATGGGATGTTTACAAGGC	569	This study
	R: GAATTCTTGCGCGGAGCGTTGAACTT		
*dxs*	F: GCTCTAGATATTAATAGGCCCCTG	1838	This study
	R: CGAATTCCAGGAGTGGAGTAGGGA		
Primers of quantitative real-time PCR			
*bla*_CTX-M_-q	F: GCCGCCGACGCTAATACA	127	This study
	R: TGCTTCCTGGGTTGTGGG		
*nusG*-q	F: TTATTTCACCGCCAAACTTCA	155	This study
	R: AAGACGGGCAGACTCCAGG		
*dxs*-q	F: CGAGAAACTGGCGATCCTTA	113	AF035440
	R: CTTCATCAAGCGGTTTCACA		
Primers of real-time relative quantitative PCR			
*repA*	F: ATTCTTGCCTGTTTGTGGTGTGCG	124	This study
	R: TTCGCTTTTCTGGCTCCTGTAC		
*repA1*	F: CGCAGCCGTGTGGAATGG	111	This study
	R: GCGGAAACGCTCACGAACAA		
*repA3*	F: GGTACAAAGCGAGCATACCGAA	117	This study
	R: CGCAGCAGGAATGAACACT		
*repA4*	F: AGAACCGAAACCACAAAGCC	122	This study
	R: GACTGACAGCGATGAAGATGT		

### Growth kinetics and plasmid stabilities

Growth curve assays were performed according to the method described in our previous study (20). The *E. coli* C600_FII_,C600_I1,_ and C600_FII+I1_ cultures grew in a tube at 37°C with shaking and diluted 1:100 into fresh media every 24 h for 336 h to investigate the stability of their plasmids (29). Viable counts were performed at 0, 48, 96, 144, 192, 240, 288, and 336 h. For every timepoint, dilutions of the cultures were plated to nonselective and cefotaxime-containing media. Cells not containing the FII plasmid were killed by cefotaxime-containing media, and cells containing I1 plasmids were verified by PCR using the prime *nusG* (a NusG family protein in plasmid I1). The stability of plasmids was calculated by the following formula : plasmids-carrying cells counts / antibiotic-free plate counts.

### *In vitro* fitness assays

A competition experiment was conducted with the strains C600_FII_, C600_I1_, and C600_FII+I1_ in fresh LB broth, following the method described in previous studies (29). Appropriately diluted samples of competition mixtures were plated onto LB agar containing 4 mg/L of cefotaxime and antibiotic-free at 0 h, 24 h, 48 h, 72 h, 96 h, 120 h, and 144 h to determine total bacterial count. Further, each colony was amplified by PCR using *nusG* primers to verify whether it carried plasmid I1. Finally, the relative fitness (RF) was calculated as previous (20). Experiments were repeated in at least three separate assays.

### Quantitative real-time PCR (qPCR)

The copy numbers of plasmids in strains C600_FII_, C600_I1_, and C600_FII+I1_ were measured by qPCR operate according to the instructions of ChamQ Universal SYBR qPCR Master Mix (Nanjing Vazyme Biotech Co., Ltd.). The genes *bla*_CTX-M_, *nusG,* and *dxs* (1-deoxy-D-xylose-5-phosphate synthase, as a housekeeping gene) were cloned into pUC19 to generate pUC19-*bla*_CTX-M_, pUC19-*nusG,* and pUC19-*dxs.* Thereafter, they were used as template DNA and *bla*_CTX-M_-q, *nusG*-q, and *dxs*-q as primers (Table 3） for qPCR amplifications, and corresponding standard curves were established. Soon afterward, the genomic DNA of strains C600_FII_, C600_I1,_ and C600_FII+I1_ was extracted using the HiPure Bacterial DNA Kit (Takara, China), and qPCR was performed with the aforementioned primers. The copy numbers of plasmids per cell were calculated as *bla*_CTX-M_/*dxs* and *nusG*/*dxs* ratios. The experiment was performed in triplicate.

### Mating assays

The donor strains C600_FII_ and C600_FII+I1_ were re-transferred to recipient *E. coli* J53 AZ^R^. Briefly, standing overnight cultures were diluted 1:200 in fresh LB broth and incubated with shaking at 37°C to an OD_600_ of 0.5. Thereafter, the donor and the recipient were mixed in pairs in a 5.0 mL total volume at a ratio of 1:1 and mated for 4 h at 37°C. Mating was stopped by cooling the samples on ice for 1 min and vortexing vigorously for 1 min. Then, each sample was serially diluted in 0.9% NaCl and plated onto LB agar supplemented with 4 mg/L cefotaxime. The plates were subsequently incubated overnight at 37°C and counted. Colonies of C600_FII+I1_ and *E. coli* J53 mixture were amplified to verify whether it co-harbored plasmids FII and I1. Each experiment was performed at least three times. Thereafter, mating efficiency was calculated and evaluated.

### Plasmid invasion assays

The plasmid invasion assay was conducted following the previous study by the Yang team with some modifications (30). Briefly, overnight cultures of *E. coli* C600 were diluted 100-fold into 2 mL of fresh antibiotic-free LB broth and mixed with the 100,000-fold dilutions of C600_FII_ or/and C600_FII+I1_ at a ratio of 1:1. The LB broth was replaced every 24 hours, and an equal portion of the samples was plated onto LB agar containing rifampin (400 µg/mL) or rifampin (400 µg/mL) +cefotaxime (4 µg/mL) to determine the viable counts after gradient dilutions. The plates were then incubated under appropriate conditions, and the viable bacterial counts were determined through gradient dilutions. All viable bacteria were able to grow on the plates contained rifampin, and bacteria containing *bla*_CTX-M_-IncFII were also able to grow on the media supplemented with rifampin +cefotaxime. Further, PCR was used to distinguish C600_FII_, C600_I1,_ and C600_FII+I1_ by the specific gene *bla*_CTX-M_ and *nusG*. All invasion assays were replicated three times biologically.

### Swimming motility assay

The single colonies C600_FII_, C600_I1,_ and C600_FII+I1_ were cultured in fresh LB broth overnight at 37°C at 180 RPM and then transferred to fresh LB broth, cultured to an OD_600_ of 0.5. Bacteria were point-inoculated in the swimming assay plate containing 0.3% agar with a sterile toothpick. Then, the plates were incubated at 37°C for 28 h. The motility ability was assessed by examining the movement distance of the bacteria away from the inoculation point (16). The experiment was performed with three biological replicates.

### Real-time relative quantitative PCR (RT-qPCR)

Based on whole-genome sequencing and comparative analysis, plasmid FII encodes the genes *repA*, *repA1*, *repA2*, and *repA4*. Therefore, the expression levels of all *rep* genes in strains C600_FII_ and C600_FII+I1_ were assayed using RT-qPCR (19). Primer sequences for RT-qPCR are listed in Table 3. A single colony of each strain was cultured in the LB medium at 37°C. After overnight growth, the cultures were diluted 1:100 in fresh LB broth and grown to an OD_600_ of 0.5. Thereafter, the total bacterial RNA was extracted using a TaKaRa MiniBEST Universal RNA Extraction Kit (TaKaRa Bio, Inc., Shiga, Japan). The quantity and purity of the extracted RNA were determined using a NanoDrop 1000 spectrophotometer (Thermo Scientific, Hvidovre, Denmark) by measuring the A_260_ absorption and calculating the A_260_/A_280_ ratio. The cDNA samples were synthesized from the extracted RNA using a cDNA reverse transcription kit with gDNA Eraser (TaKaRa Bio, Inc.). RT-qPCR was then performed according to our previous study (20). The relative expression levels in C600_FII+I1_ compared to C600_FII_ were calculated using the 2^−ΔΔCT^ method. Three independent biological replicates were conducted for each experiment.

### Intracellular adenosine triphosphate (ATP) levels

Intracellular ATP levels of strains C600_FII_, C600_I1_, and C600_FII+I1_ were assessed using an Enhanced ATP Assay Kit (Beyotime, Shanghai, China). Overnight cultures of strains were removed and resuspended to obtain an OD_600_ of 0.5. Bacterial pellets were lysed by lysozymes, and the luminescence of the supernatant was determined using the Spark 10 M Microplate reader (Tecan). The experiment was performed at least in triplicate.

### Statistical analyses

The statistical analyses were performed with GraphPad Prism v8.0.2 (GraphPad Software). Data were presented as mean ± standard deviation (SD) of three replications. Mean values between groups were compared using unpaired *t*-test, and *P* < 0.05 was considered to be statistically significant.

## AFTER EPUB

*[This article was published on 30 April 2024 with an error in the introduction. The introduction was corrected in the current version, posted on 13 May 2024.]*
